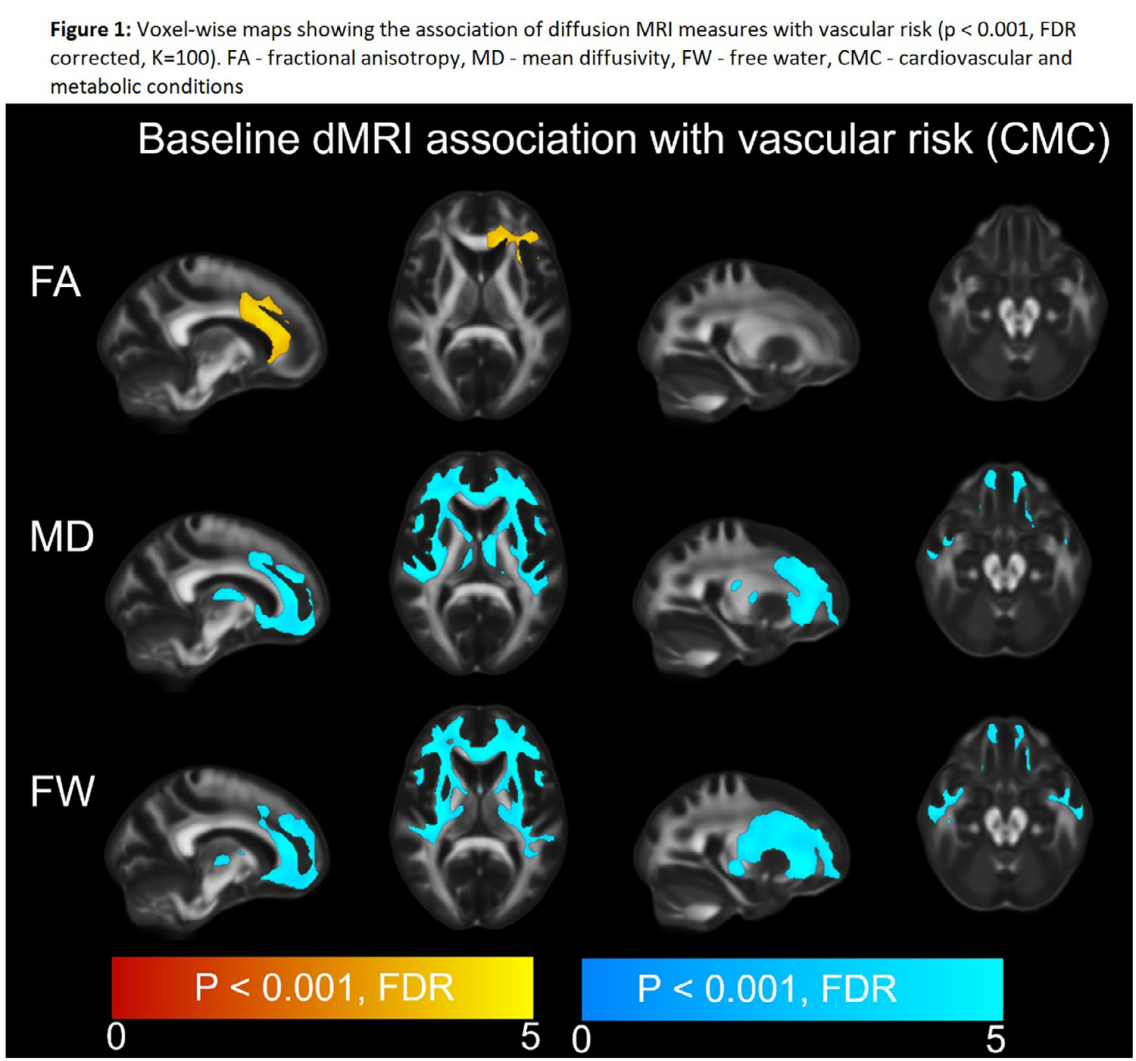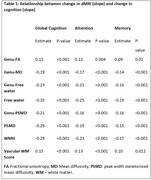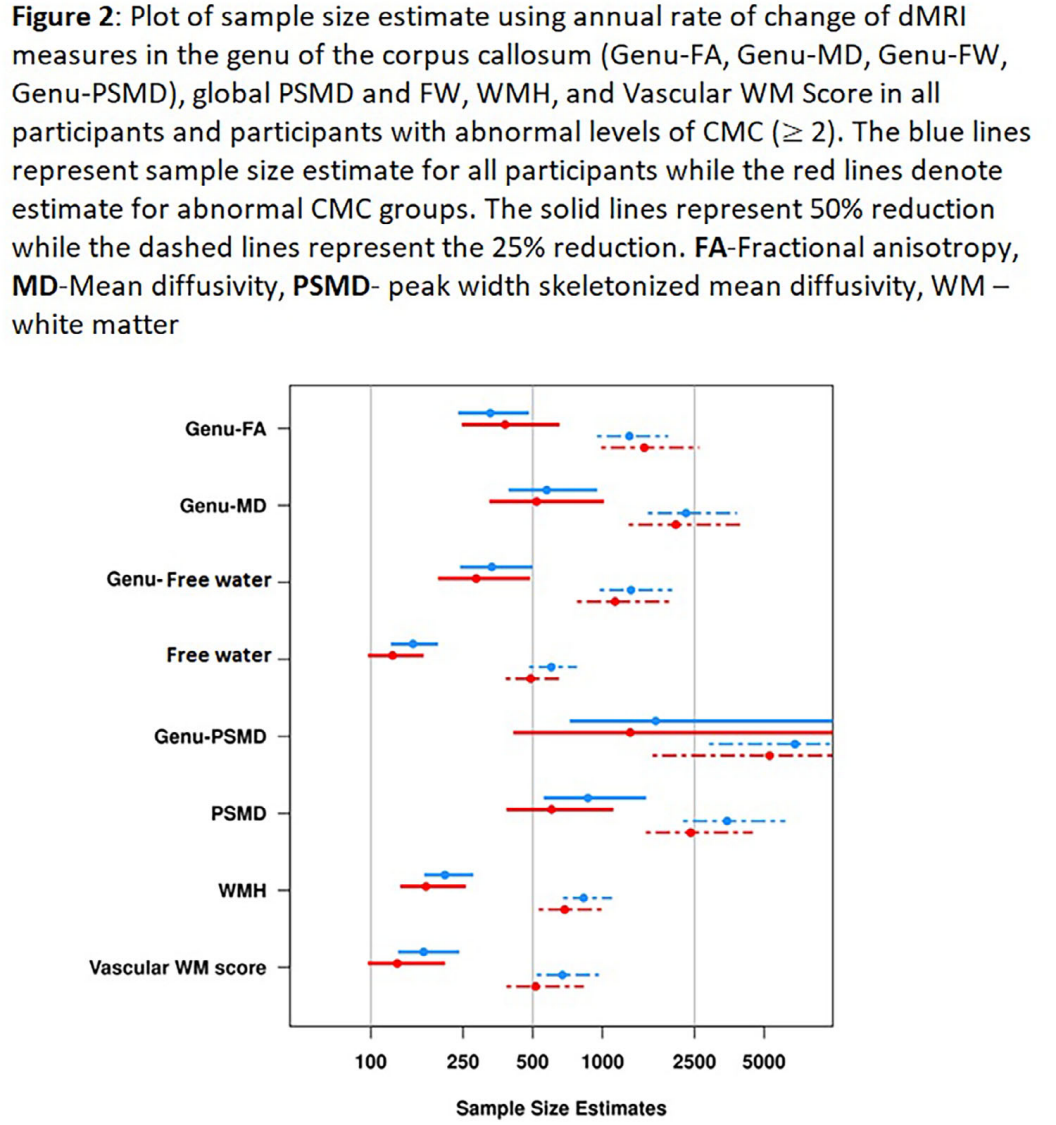# Comparing regional and global diffusion MRI markers as biomarkers of VCID

**DOI:** 10.1002/alz.090097

**Published:** 2025-01-09

**Authors:** Sheelakumari Raghavan, Robert I. Reid, Scott A. Przybelski, Michael G. Kamykowski, Timothy G. Lesnick, Jonathan Graff‐Radford, Val J. Lowe, David S. Knopman, Clifford R. Jack, Ronald C. Petersen, Prashanthi Vemuri

**Affiliations:** ^1^ Mayo Clinic, Rochester, MN USA; ^2^ Department of Quantitative Health Sciences, Mayo Clinic, Rochester, MN USA; ^3^ Mayo Clinic, Quantitative Health Sciences, Rochester, MN USA; ^4^ Department of Neurology, Mayo Clinic, Rochester, MN USA; ^5^ Department of Radiology, Mayo Clinic, Rochester, MN USA; ^6^ Mayo Clinic Alzheimer's Disease Research Center, Rochester, MN USA; ^7^ U.S. Advisory Council on Alzheimer’s Research, Care, and Services, Washington, DC USA

## Abstract

**Background:**

Quantifying white matter using diffusion MRI (dMRI) has been proposed for measuring early microstructural tissue changes due to cerebral small vessel disease and aid in quantifying vascular contributions to cognitive impairment and dementia (VCID). Our goal was to compare the usefulness of longitudinal white matter changes in the commonly available diffusion MRI measures for VCID prevention trials.

**Method:**

We included 718 participants over 50 years of age (mean age: 71.1(9.6) years) from the Mayo Clinic Study of Aging, a population‐based sample, with at least two dMRI scans and structural imaging. We computed the commonly available single‐shell dMRI measures (fractional anisotropy, mean diffusivity, and two MarkVCID measures ‐ free water and peak‐width skeletonized mean diffusivity [PSMD]) at each time point. We tested for voxel‐wise associations between dMRI markers and vascular risk measured by cardiovascular metabolic condition (CMC) and observed a region‐specific dependance across all dMRI measures. Using both global and regional dMRI measures, we (i) examined the longitudinal association of dMRI measures with cognition and (ii) computed sample size estimates for a hypothetical clinical trial. We also included white matter hyperintensities (WMH) and our previously proposed composite vascular white matter score (combination of WMH and fractional anisotropy of the genu) as a comparison.

**Result:**

Vascular risk was associated with all single shell dMRI measures in the genu of the corpus callosum, which we included as a regional dMRI marker for comparison (Figure 1). All dMRI markers correlated with cognitive performance longitudinally (Table 1) and had comparable sample size estimates required for hypothetical VCID clinical trials (Figure 2). Further, global free water and the composite vascular white matter score had the smallest sample size estimates.

**Conclusion:**

All commonly used dMRI markers had significant frontal lobe changes due to vascular risk. Both global and regional corpus callosum dMRI markers were sensitive to longitudinal cognitive decline, suggesting their utility in measuring the slowing down of VCID. The composite vascular white matter score, global free water, and WMH show promise as VCID biomarkers. Further work is needed to validate these markers on multiple populations.